# Low Temperature Thermal Treatment of Incineration Fly Ash under Different Atmospheres and Its Recovery as Cement Admixture

**DOI:** 10.3390/ma16113923

**Published:** 2023-05-24

**Authors:** Tingshu He, Jiangbo Li, Xiaodong Ma, Yongqi Da, Hudie Yuan

**Affiliations:** College of Materials Science and Engineering, Xi’an University of Architecture and Technology, Xi’an 710055, China; 17791557791@163.com (Y.D.); 15735044352@163.com (H.Y.)

**Keywords:** water-washed incineration fly ash, thermal treatment, carbon sequestration, dioxin degradation, cement admixture

## Abstract

Municipal solid waste incineration fly ash is classified as hazardous waste because it contains dioxins and a variety of heavy metals. It is not allowed to be directly landfilled without curing pretreatment, but the increasing production of fly ash and scarce land resources has triggered consideration of the rational disposal of fly ash. In this study, solidification treatment and resource utilization were combined, and the detoxified fly ash was used as cement admixture. The effects of thermal treatment in different atmospheres on the physical and chemical properties of fly ash and the effects of fly ash as admixture on cement properties were investigated. The results indicated that the mass of fly ash increased due to the capture of CO_2_ after thermal treatment in CO_2_ atmosphere. When the temperature was 500 °C, the weight gain reached the maximum. After thermal treatment (500 °C + 1 h) in air, CO_2_, and N_2_ atmospheres, the toxic equivalent quantities of dioxins in fly ash decreased to 17.12 ng TEQ/kg, 0.25 ng TEQ/kg, and 0.14 ng TEQ/kg, and the degradation rates were 69.95%, 99.56%, and 99.75%, respectively. The direct use of fly ash as admixture would increase the water consumption of standard consistency of cement and reduce the fluidity and 28 d strength of mortar. Thermal treatment in three atmospheres could inhibit the negative effect of fly ash, and the inhibition effect of thermal treatment in CO_2_ atmosphere was the best. The fly ash after thermal treatment in CO_2_ atmosphere had the possibility of being used as admixture for resource utilization. Because the dioxins in the fly ash were effectively degraded, the prepared cement did not have the risk of heavy metal leaching, and the performance of the cement also met the requirements.

## 1. Introduction

With accelerated urbanization and the improvement of people’s living standards, the production of municipal solid waste (MSW) has been increasing [1,2]. Incineration and landfill are the two main disposal methods of MSW in China [3,4]. After MSW incineration (MSWI) treatment, the volume of waste can be significantly reduced by approximately 85~90%, the mass by 60~90%, and the organic matter by nearly 100% [5]. The increase in the production of MSW will naturally lead to an increase in the amount of incineration, and the incineration method plays an increasingly important role in the field of MSW treatment with its high efficiency and excellent reduction effect.

MSWI fly ash (MSWI FA) is a kind of particle by-product that is produced after MSWI. It refers to the mixed particulate matter collected by the flue gas from the incineration furnace after being treated by the air pollution control (APC) device [6]. The MSWI FA generation usually accounts for about 3%~15% of MSW amount depending on different MSWI furnaces [7]. However, MSWI FA is classified as hazardous waste due to its large number of toxic substances such as leachable heavy metals and dioxins (PCDD/Fs). Therefore, proper disposal for MSWI FA is crucial.

In China, MSWI FA is mainly landfilled after solidification/stabilization with cementitious materials and chelating agents [8,9]. However, due to limited land resources, the construction of landfills is becoming more and more difficult. Landfill treatment is increasingly inconsistent with China’s sustainable development strategy, and more importantly, PCDD/Fs cannot be degraded. MSWI FA has potential pozzolanic activity due to being rich in Ca, Si, Al, and other elements, and relatively small particles ensure that MSWI FA has sufficient surface area for chemical reaction [10,11]. These physical and chemical properties give the possibility of MSWI FA incorporation into cement-based materials. However, the existence of heavy metals, dioxins, and chlorides in MSWI FA seriously limits its direct utilization, and it needs to be properly treated to realize the resource utilization of MSWI FA. Heat treatment has received the most extensive attention due to its excellent performance in the solidification of heavy metals, the decomposition and destruction of dioxins, and other toxic organic compounds, and the production of environmentally non-toxic reuse materials [12,13]. It has been shown that PCDD/Fs in MSWI FA can be effectively degraded at high temperature, and after melting treatment, MSWI FA becomes a stable and non-toxic resource [14,15]. However, MSWI FA contains a large amount of catalytic metals, organic matter, carbon source, and chlorine source, which makes it easy to synthesize PCDD/Fs again in the temperature range of 200~450 °C [16]. Chen et al. reported that high temperature did lead to the decomposition of PCDD/Fs, but it also greatly promoted the migration of substances that formed PCDD/Fs from MSWI FA to flue gas, which might cause a large reorganization of PCDD/Fs in the low temperature zone [17]. The catalytic metals (such as iron, copper, chromium, and zinc) in MSWI FA were more important than chlorine or carbon sources to promote the recombination of PCDD/Fs [17]. Therefore, reducing the volatilization of heavy metals by reducing the disposal temperature of MSWI FA is beneficial to avoid the recombination of PCDD/Fs, and high temperature will inevitably increase energy consumption and treatment cost. Based on these problems, low temperature thermal treatment of MSWI FA has received more attention.

Wu et al. found that PCDD/Fs in fly ash are easy to decompose above 350 °C in nitrogen atmosphere, and their decomposition pathways include dechlorination and destruction of ring structure, and PCDD/Fs are rarely desorbed into flue gas [18]. It has also been reported that as long as the treatment time is long enough, it is also beneficial to destroy dioxins at about 300 °C or even 260 °C [19,20]. In low temperature thermal treatment, the treatment temperature is often more important than time [21]. When the temperature rises, the decomposition efficiency will be more significant. Therefore, low temperature thermal treatment technology is a very promising fly ash dioxin degradation technology. In addition, MSWI FA has certain carbon dioxide storage potential [22,23]. Sun et al. conducted a gas–solid carbonation experiment of waste incineration fly ash and pure carbon dioxide at 200~500 °C, the maximum calcium conversion rate was 57%, and each kilogram of solid waste could store 0.12 kg of carbon dioxide [22]. After carbonation, the leaching of heavy metals in MSWI FA also decreased to a certain extent, because most of the heavy metals were bound to carbonate after carbonation reaction, from the exchangeable part to the carbonized part, becoming more stable [24,25]. However, whether PCDD/Fs in carbonized fly ash was decomposed and whether the treated fly ash can be used as a resource was rarely investigated in previous studies.

Because cement solidification technology can effectively reduce the environmental pollution caused by MSWI FA, especially significantly reducing the leaching concentration of heavy metals [26], and fly ash has a certain pozzolanic activity, it will be a good disposal method to add fly ash after detoxification modification pretreatment to cement-based materials for resource utilization. In this study, the MSWI FA used was pre-treated with water to wash out chlorine, and then the water-washed MSWI FA was subjected to low-temperature thermal treatment under different atmospheres (air, carbon dioxide, and nitrogen). In particular, thermal treatment under carbon dioxide atmosphere was the focus of this study, because if the water-washed MSWI FA could absorb carbon dioxide while modifying, it could appropriately reduce the current excess carbon dioxide. The mass, particle size distribution, phase composition, microstructure, heavy metal leaching characteristics, and dioxin content of the water-washed MSWI FA after treatment in different atmospheres were compared. Following that, the treated water-washed MSWI FA was used as cement admixture to explore its effect on cement properties, including water consumption of standard consistency, setting time, mortar fluidity, strength, and hydration products. In addition, the leaching characteristics of heavy metals in the prepared cement were evaluated. Through the above, the feasibility of using the treated water-washed MSWI FA as cement admixture was demonstrated. The research results are helpful to broaden the resource utilization of MSWI FA.

## 2. Materials and Experiment

### 2.1. Materials

**Figure 1:**
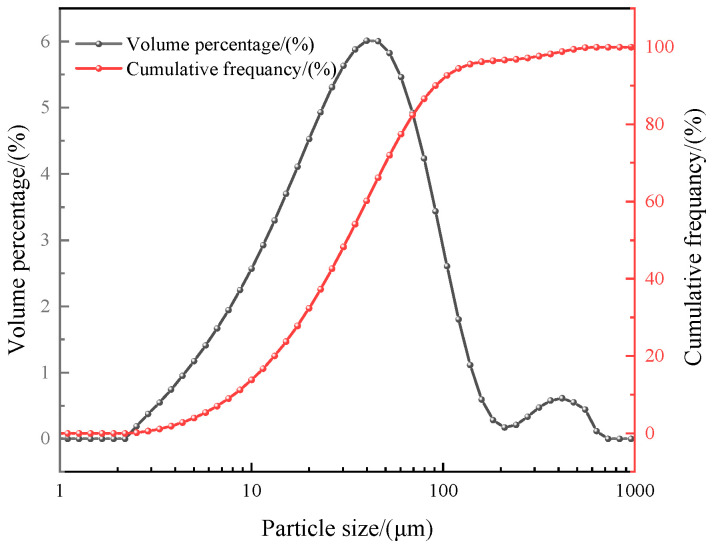
Particle size distribution of water-washed MSWI FA.

In this study, the water-washed MSWI FA was provided by Anhui Conch Environmental Protection Group Co., Ltd., Wuhu, China. The obtained water-washed MSWI FA was dried to constant weight at 60 °C, homogenized by a quartering method, and finally transferred to a closed container for use. The density of water-washed MSWI FA was 2.24 g/cm^3^, and the BET specific surface area was 23.18 m^2^/g. The chemical composition was measured by X-ray fluorescence analysis (XRF, PANalytical Axios, PANalytical B.V., Almelo, The Netherlands), and the results are shown in Table 1. The main components of the incineration fly ash were calcium oxide (CaO, 48.21%), sulfur trioxide (SO_3_, 8.54%), and silicon dioxide (SiO_2_, 6.59%), and it had a high loss on ignition of 19.59%. The contents of heavy metals are shown in Table 2, and the results presented the contents of zinc (Zn), plumbum (Pb), cadmium (Cd), and copper (Cu) were as high as 3212.19 mg/L, 312.95 mg/L, 133.80 mg/L, and 116.07 mg/L. In addition, the contents of arsenic (As), chromium (Cr), antimony (Sb), and nickel (Ni) were as low as 74.16 mg/L, 72.68 mg/L, 68.96 mg/L, and 51.46 mg/L. The particle size distribution is shown in Figure 1. The incineration fly ash had a fine particle size of D10 = 8.10 μm, D50 = 31.58 μm, and D90 = 90.36 μm.

The raw materials also included Portland cement clinker and natural dihydrate gypsum, which were provided by Shaanxi Yaobai Special Cement Co., Ltd., Xi’an, China. The chemical composition is shown in Table 3. The density of cement clinker was 3.07 g/cm^3^ and the content of f-CaO was 1.01%. Standard sand was produced by Xiamen Aisiou Standard Sand Co., Ltd., Xiamen, China.

### 2.2. Experiment

#### 2.2.1. Low Temperature Thermal Treatment of the Water-Washed MSWI FA

The water-washed MSWI FA was heat-treated at different atmospheres (air, carbon dioxide, and nitrogen) in a high temperature-controlled atmosphere test furnace (QFL-16-8, Luoyang Antler Instrument Co., Ltd., Luoyang, China). The gas flow rate was 60 mL/min, the heating rate was 5 °C/min, and after reaching the target temperature, the holding time was 1 h, and the sample was taken out after falling to room temperature. The concentration of nitrogen and carbon dioxide atmosphere used was greater than 99%. Following that, the mass change, particle size distribution, phase composition, microstructure, heavy metal leaching characteristics, and dioxin content of the treated water-washed MSWI FA were tested to determine the modification of incineration fly ash by low temperature thermal treatment.

#### 2.2.2. Preparation of Cement with Different Admixture

In the light of Chinese standard GB 175-2007 (Common Portland cement), firstly, the P•I Portland cement was ground by a ball mill (RK/ZQM (BM), Wuhan Rock Grinding Equipment Manufacturing Co., Ltd., Wuhan, China) as the control group, and the dosage of water-washed MSWI FA in the control group was 0. The water-washed MSWI FA after heat treatment at 500 °C in air (denoted as W1), carbon dioxide (denoted as W2) and nitrogen (denoted as W3) atmospheres and the untreated water-washed MSWI FA (denoted as W0) were selected to replace the Portland cement clinker with different dosages (5%, 10%, and 15% based on the weight percentage) to prepare P•O Portland cement. The Blaine specific surface area of the prepared cement was controlled to be (350 ± 10) m^2^/kg. The formula of the prepared cement is shown, in Table 4.

#### 2.2.3. Determination of Dioxin Content

Dioxins in the incineration fly ash were determined according to the Chinese standard HJ 77.3-2008 (Solid waste; determination of polychlorinated dibenzo-p-dioxins and polychlorinated dibenzofurans; isotope dilution HRGC-HRMS). The instrument used was a high-resolution gas chromatography-high-resolution dual-focus magnetic mass spectrometer (Trace GC Ultra, Thermo Fisher Scientific, Waltham, MA, USA).

#### 2.2.4. Determination of the Contents of Heavy Metals

The determination of heavy metal content required the digestion of heavy metal elements in incineration fly ash. The digestion method was referred to Chinese standard HJ 832-2017 (Soil and sediment; digestion of total metal elements; microwave-assisted acid digestion method). The concentration of heavy metals in the liquid after digestion was determined by inductively coupled plasma-mass spectrometry (ICP-MS, Aglient 7800, NYSE: A, Palo Alto, CA, USA).

#### 2.2.5. Leaching Characteristics of Heavy Metals

The leaching toxicity of the incineration fly ash and the prepared cement was investigated by the tests according to the Chinese standard HJ 557–2010 (Solid waste; extraction procedure for leaching toxicity; horizontal vibration method) and HJ/T 300-2007 (Solid waste; extraction procedure for leaching toxicity; acetic acid buffer solution method). After leaching, the leaching concentrations of heavy metals in samples under different leaching environments were determined by ICP-MS, and then the leaching risk was evaluated according to the Chinese standard GB 16889-2008 (Standard for pollution control on the landfill site of municipal solid waste). When measuring the leaching characteristics of heavy metals in cement, cement mortar specimens with standard curing for 28 days were selected, and the standard curing condition was that the temperature was (20 ± 1) °C and the relative humidity was not less than 90%.

#### 2.2.6. Particle Size Distribution, XRD, and SEM

The particle size distribution of water-washed MSWI FA before and after treatment was measured by a laser particle size analyzer (Malvern Mastersizer 2000, Malvern, UK), and the particle size range was 0.02~2000 μm. X-ray diffraction (XRD, X’Pert PRO MPD, PANalytical B.V., Almelo, The Netherlands) was used to analyze the phase composition of the incineration fly ash before and after treatment and cement hydration products. The working conditions were Cu target Kα line at 40 kV and 40 mA, and the scanning speed was set as 5°/min (2θ range: 5°–70°). Scanning electron microscopy (SEM, Zeiss Sigma 300, Carl Zeiss AG, Oberkochen, Germany) was used to observe the microstructure of the incineration fly ash before and after treatment.

#### 2.2.7. Cement Workability and Mortar Fluidity

The water consumption of standard consistency and setting time of the prepared cement were measured according to the Chinese standard GB/T 1346-2011 (test methods for water requirement of normal consistency, setting time, and soundness of the Portland cement). The fluidity of mortar was measured according to Chinese standard GB/T 2419-2005 (test method for fluidity of cement mortar).

#### 2.2.8. Mechanical Properties of Cement

According to the Chinese standard GB/T 17671-2021 (Test method of cement mortar strength), cement mortars with a water to cement weight ratio of one-half and a cement to standard sand weight ratio of one-third were prepared. After stirring evenly according to the prescribed procedure, a mold with a size of 40 mm × 40 mm × 160 mm was loaded and placed in a standard curing room (temperature of (20 ± 1) °C, relative humidity of not less than 90%) for 24 h. Following that, the mold was removed, and the mortar specimens continued to be cured to the corresponding age. The flexural and compressive strengths were measured at the curing age of 3 d and 28 d. The mechanical properties of cement mortar specimens were measured by automatic cement flexural and compressive machine (YAW-SERIES, Zhejiang Lixian Test Instrument Manufacturing Co., Ltd., Shaoxing, China). The loading rate of flexural strength was (50 ± 10) N/s. The average value of the three specimens was taken as the flexural strength, and the standard deviation was calculated. The loading rate of compressive strength was (2400 ± 200) N/s. The average value of the six specimens was taken as the compressive strength, and the standard deviation was calculated.

#### 2.2.9. Preparation of Cement Hydration Products for XRD Analysis Samples

Cement paste with a water–cement ratio of one-half was prepared. After standard curing to the corresponding age, it was crushed into small pieces, immersed in anhydrous ethanol for 3 days to terminate hydration, and then dried in an oven at 40 °C for 48 h. The small pieces were ground through a 75 μm sieve for XRD analysis.

## 3. Results and Discussion

### 3.1. Effect of Thermal Treatment Atmosphere on Physicochemical Properties of the Water-Washed MSWI FA

#### 3.1.1. The Mass Change

**Figure 2:**
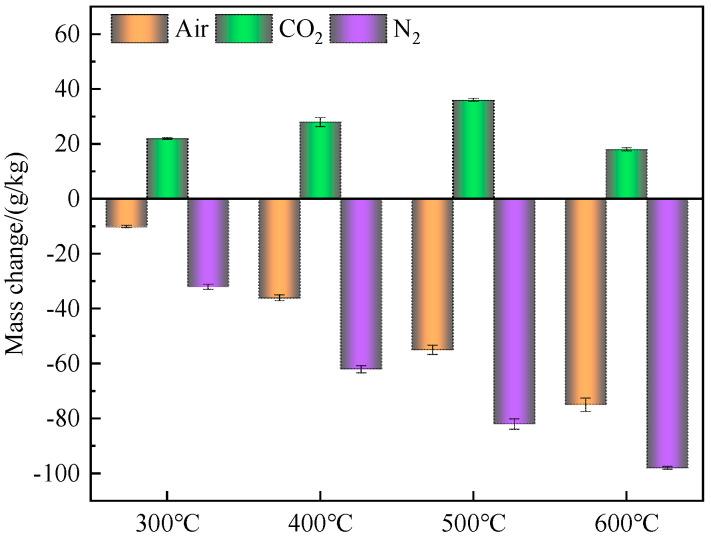
Mass changes of incineration fly ash after thermal treatment under different conditions.

The mass changes of water-washed MSWI FA after thermal treatment under different conditions are shown in Figure 2. From the diagram, it can be seen that the mass of incineration fly ash was reduced under air and N_2_ atmosphere, and the mass loss rate increased with the increase of temperature. At the same temperature, the mass loss of incineration fly ash in N_2_ atmosphere was greater than that in air atmosphere, because there was a part of CO_2_ in the air, which had a carbonation reaction with incineration fly ash. The mass of incineration fly ash increased after heat treatment in CO_2_ atmosphere, and the weight gain reached the maximum at 500 °C. This was because some calcium carbonate decomposed at 600 °C and released the sealed CO_2_, which proved that incineration fly ash had the ability to fix CO_2_.

Tian et al. used thermo-gravimetric analysis to determine the ability of air pollution control residues (APCr) to capture CO_2_, and the calculation method was that the CO_2_ absorption measured by thermo-gravimetric analysis was the sum of the weight gain of dry APCr in CO_2_ atmosphere and its weight loss in N_2_ under the same conditions [27]. According to this calculation method, the incineration fly ash was heat-treated at 500 °C in CO_2_ atmosphere, and the weight gain was 36 g/kg. The incineration fly ash was heat-treated at 500 °C in N_2_ atmosphere, and the mass loss was 82 g/kg. Therefore, the actual CO_2_ absorption of the incineration fly ash was 118 g/kg, which was the largest CO_2_ absorption in this paper. Therefore, the effect of heat treatment atmosphere on the physical and chemical properties of water-washed MSWI FA was studied at 500 °C.

#### 3.1.2. Particle Size Distribution

**Figure 3:**
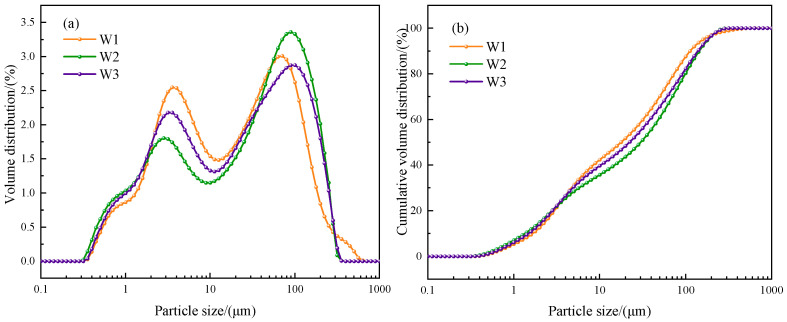
The particle size distribution of incineration fly ash after heat treatment in different atmospheres. (**a**) Volume distribution, (**b**) Cumulative volume distribution.

The particle size distribution of incineration fly ash after heat treatment in different atmospheres is shown in Figure 3, and Table 5 shows the key parameter of particle size distribution. From Figure 1, it can be seen that the particle size of incineration fly ash was generally unimodal distribution, the distribution range was mainly 8.10~90.36 μm, and the average particle size was 31.58 μm. After heat treatment in different atmospheres, the particle size of fly ash showed asymmetric bimodal distribution, which might be due to the partial sintering of particle edges and the agglomeration of some particles during heat treatment. The distribution range was mainly 1.66~8.93 μm and 25.18~112.81 μm in air atmosphere, 1.35~7.10 μm and 25.18~144.10 μm in CO_2_ atmosphere, and 1.48~7.10 μm and 25.18~138.74 μm in N_2_ atmosphere. The average particle size of fly ash decreased to 17.78 μm, 30.00 μm, and 22.21 μm after heat treatment in air, CO_2_, and N_2_ atmospheres, respectively. It could also be seen from Figure 3 that the volume distribution of particle size in the first peak of the particle size volume distribution of the fly ash after heat treatment in CO_2_ atmosphere was the smallest, and the volume distribution of particle size in the second peak becomes the largest. This might be due to the heat treatment of fly ash in CO_2_ atmosphere. The surface of the particles was only partially sintered, and some CaCO_3_ crystals were generated. After heat treatment in the three atmospheres, the dispersion of fly ash particle size distribution in CO_2_ atmosphere was the smallest. Dispersion was used to describe the dispersion degree of particle size distribution. It was calculated by Equation (D90 − D10)/D50. The smaller the dispersion was, the more uniform the particle size distribution was.

#### 3.1.3. Phase Composition

**Figure 4:**
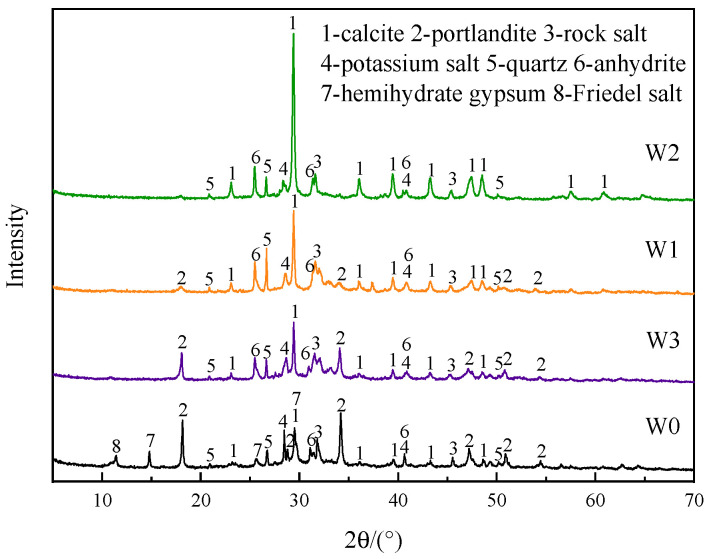
XRD analysis results of fly ash after heat treatment under different atmospheres.

The XRD analysis results of fly ash before and after heat treatment under different atmospheres are shown in Figure 4. The main mineral phases contained in untreated fly ash were: calcite (CaCO_3_), portlandite (Ca(OH)_2_), rock salt (NaCl), potassium salt (KCl), quartz (SiO_2_), anhydrite (CaSO_4_), hemihydrate gypsum (CaSO_4_·0.5H_2_O), and Friedel salt (3CaO·Al_2_O_3_·CaCl_2_·10H_2_O). These chlorine-containing substances were mainly the product of the reaction of HCl in the flue gas with lime and other substances [28]. Friedel salt was the main insoluble chloride salt in fly ash [29].

After heat treatment at 500 °C, the diffraction peaks of CaSO_4_·0.5H_2_O and Friedel salt disappeared in all three atmospheres, while the diffraction peak intensity of CaSO_4_ was enhanced, indicating that CaSO_4_·0.5H_2_O was dehydrated and transformed into CaSO_4_ when the temperature rose to 500 °C. Friedel salt might decompose to other substances. In addition, the diffraction peak intensity of CaCO_3_ was the highest after heat treatment in CO_2_ atmosphere, and the diffraction peak of Ca(OH)_2_ almost disappeared. This proved that almost all Ca(OH)_2_ in fly ash was carbonized to CaCO_3_, that was, Ca(OH)_2_ reacted with CO_2_ to form CaCO_3_. After heat treatment in air atmosphere, some Ca(OH)_2_ was also carbonized to CaCO_3_, while the diffraction peaks of CaCO_3_ and Ca(OH)_2_ after heat treatment in N_2_ atmosphere did not change significantly. The change of phase composition of fly ash also proved that it had carbon sequestration abilities.

#### 3.1.4. Microstructure

**Figure 5:**
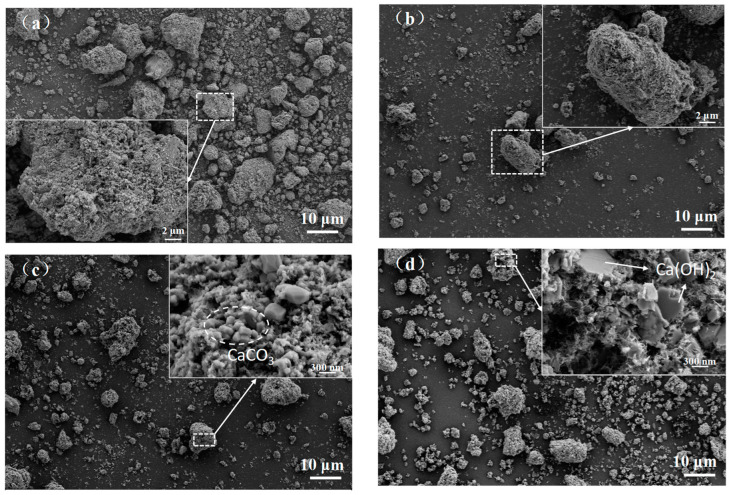
The micro-morphology of fly ash after heat treatment in different atmospheres. (**a**) W0; (**b**) W1; (**c**) W2; (**d**) W3.

The microstructures of fly ash before and after heat treatment in different atmospheres are shown in Figure 5. It can be seen in Figure 5a that the particle size of the water-washed incineration fly ash was different, the particles were randomly distributed, most of them were irregular, and only a few particles were spherical. Obviously, it could be seen that a number of particles of different shapes were stacked together. Specifically, small blocks, approximately spherical and plate-like substances, were condensed into agglomerates, interspersed with needle-like substances. There were a large number of pores inside the large particles. There were some small particles attached to the dense pores, and many irregular small particles were also stacked outside, resulting in an extremely rough surface and in a large specific surface area of the water-washed incineration fly ash.

In the low-magnification diagram, after heat treatment in three atmospheres, the fly ash particles were still of different sizes and the particle size distribution range was also wide, which was consistent with the conclusion drawn in Section 3.1.2. However, compared with the microstructure of fly ash, the irregular morphology of fly ash particles after heat treatment was significantly reduced. In the high magnification diagram, it could also be clearly observed from Figure 5b that after the heat treatment of fly ash in air atmosphere, the surface pores became less and the density increased, which might be due to the partial melting of the surface of fly ash particles during the sintering process to block the holes on the surface. It can be seen in Figure 5c that after heat treatment in CO_2_ atmosphere, a large number of calcium carbonate crystals were formed on the surface of fly ash particles. The crystals were well-formed, the surface was smooth, and the calcium carbonate crystals were closely packed together. In Figure 5d, multiple hexagonal plate-like Ca(OH)_2_ crystals were observed as being interspersed on the surface of fly ash particles, which proved that fly ash still had a large amount of Ca(OH)_2_ after heat treatment in N_2_ atmosphere, which was consistent with the XRD analysis results in Section 3.1.3.

#### 3.1.5. Heavy Metal Leaching Characteristics

The heavy metal leaching characteristics of fly ash before and after heat treatment in different atmospheres were tested by horizontal oscillation method and acetic acid buffer solution method. At the same time, the heavy metal leaching concentration limit of fly ash in the Chinese standard GB 16889-2008 was compared. The results are shown in Table 6 and Table 7. In the neutral environment, the leaching concentrations of heavy metals in the water-washed incineration fly ash did not exceed the limits specified in the standard, and the leaching concentrations of Cr and Zn were relatively large. In a weakly acidic environment, the leaching concentration of Pb in fly ash was greater than the limit (0.25 mg/L) specified in the standard, exceeding 27.48%, indicating that the water-washed incineration fly ash had a certain risk of heavy metal leaching. In addition, in the weak acid environment, the leaching concentration of Zn was the highest, which was as high as 11.3309 mg/L, which was related to the highest content of Zn in water-washed incineration fly ash. Although it did not exceed the specified limit (100 mg/L), it still had a certain leaching risk.

In the neutral environment, the leaching concentration of heavy metals in fly ash after heat treatment in three atmospheres also did not exceed the landfill limit specified in the standard, and the leaching concentration of Zn was the highest. In a weakly acidic environment, the leaching concentration of Pb in fly ash after heat treatment in air and N_2_ atmosphere was still greater than the limit value (0.25 mg/L), exceeding 15.16% and 7.92%, respectively. It could be found that compared with the leaching concentration of heavy metals in fly ash, except for Cu, the heat treatment in three atmospheres would reduce the leaching concentration of heavy metals. It had been reported that the precipitation of calcite in the pores of incineration fly ash after carbonation would increase the compactness of fly ash particles and affect the release of heavy metals [23]. Therefore, after heat treatment, it could be seen from Figure 5 that the particles of fly ash became denser, which might be the reason for the decrease of heavy metal leaching concentration.

#### 3.1.6. Dioxin Content

The toxic equivalent quantities (TEQ) of fly ash before and after heat treatment in different atmospheres are shown in Table 8, TEF meant toxic equivalent factor, and the TEQ of dioxin isomers could be obtained by multiplying the TEF of dioxin isomers by their concentration. The TEQ of fly ash was obtained by adding TEQ of 17 dioxin isomers. The TEQ of fly ash was 56.97 ng TEQ/kg. The largest contribution to the toxicity of fly ash was 2,3,4,7,8-P_5_CDF, and the corresponding TEQ was 28 ng TEQ/kg, accounting for 49.15% of the total TEQ.

After heat treatment at 500 °C in air, CO_2_ and N_2_ atmospheres, the toxic equivalent quantities were 17.12 ng TEQ/kg, 0.25 ng TEQ/kg, and 0.14 ng TEQ/kg, and the degradation rates were 69.95%, 99.56%, and 99.75%, respectively. The heat treatment under CO_2_ and N_2_ atmospheres achieved higher degradation rates. The heat treatment under air atmosphere might be due to the recombination of PCDD/Fs in the presence of oxygen [30], resulting in a lower degradation rate. The largest contribution to the toxicity of fly ash after heat treatment in CO_2_ and N_2_ atmosphere was 2,3,7,8-T_4_CDD and 1,2,3,7,8-P_5_CDD, while the largest contribution to the toxicity of fly ash after heat treatment in air atmosphere was 2,3,4,7,8-P_5_CDF, which might also be the reason for the different degradation rates.

In addition, according to the review guidelines for cement kiln co-processing of hazardous waste business license issued by the Ministry of Environmental Protection of China, the content of dioxins in the hazardous waste added to the cement mill as an alternative mixture should be less than 10 ng TEQ/kg. After the heat treatment in CO_2_ and N_2_ atmosphere, the toxic equivalent quantity of fly ash was far less than 10 ng TEQ/kg, which met the requirement, but it still could not meet the requirement after heat treatment in air atmosphere.

### 3.2. The Effect of Water-Washed Incineration Fly Ash before and after Heat Treatment as Admixture on Cement Properties

#### 3.2.1. Water Consumption of Standard Consistency

**Figure 6:**
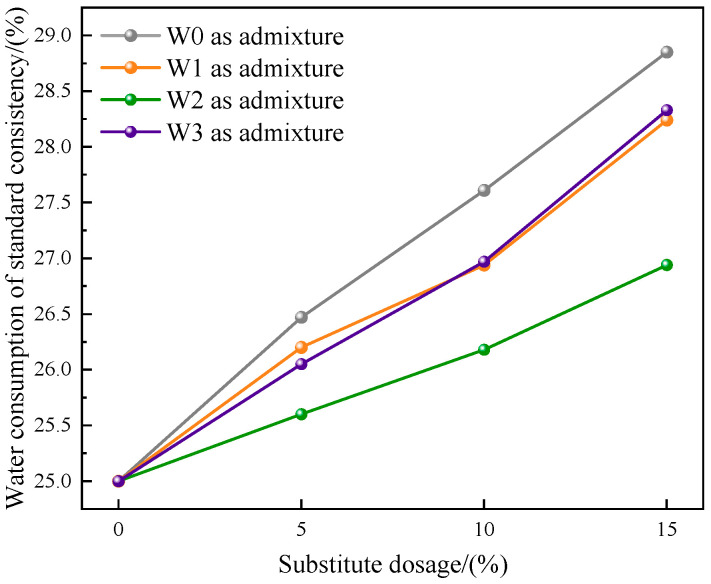
Water consumption of standard consistency of the prepared cement.

The results of the water consumption of standard consistency of cement prepared by different admixtures are shown in Figure 6. The water consumption of standard consistency referred to the water consumption required when the cement was mixed into a specific plastic state. The results were expressed as a percentage of cement quality.

Compared with the control group, the addition of water-washed incineration fly ash would increase the water consumption of standard consistency of cement, and the water consumption of standard consistency gradually increased with the increase of dosage. This was closely related to the physical and chemical properties of fly ash. The surface of fly ash particles was rough and porous, and the distribution of pores was more, resulting in strong hygroscopicity, and water molecules would be adsorbed inside the pores. Under the same dosage, the water consumption of standard consistency of cement prepared by the untreated fly ash as admixture was greater than that of cement prepared by fly ash as admixture after heat treatment in three atmospheres, and the water consumption of standard consistency of cement prepared by fly ash used as admixture after heat treatment in CO_2_ atmosphere was the smallest. It could be concluded that the heat treatment under CO_2_ atmosphere had a better improvement effect on the problem of increasing water consumption of cement standard consistency caused by incorporation of the untreated fly ash. The reason might be that after heat treatment in CO_2_ atmosphere, calcium carbonate crystals were formed on the surface of fly ash particles. The crystal surface was smooth and closely packed together, filling some pores and reducing the adsorption capacity of water. Therefore, the water consumption of standard consistency of the prepared cement was the smallest.

#### 3.2.2. Setting Time

**Figure 7:**
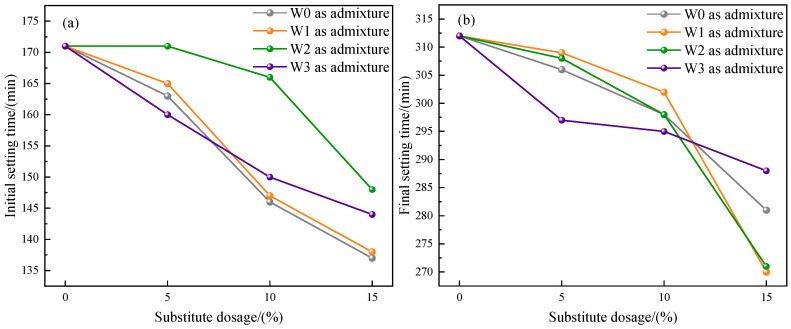
Setting time of the prepared cement. (**a**) Initial setting time; (**b**) Final setting time.

The results of setting time of cement prepared by different admixtures are shown in Figure 7, including initial setting time and final setting time. The initial setting time of the control group was 171 min, and the final setting time was 312 min. After adding water-washed incineration fly ash, the setting time of cement was shortened, and with the increase of dosage, the setting time of cement showed a downward trend. This might be due to the fact that the chloride salt in the fly ash accelerated the hydration reaction [31]. The setting time of cement prepared by using fly ash as admixture before and after heat treatment had little change. In addition, the setting time of the prepared cement met the requirements of ordinary Portland cement—initial setting time was not less than 45 min and final setting time was not more than 600 min.

#### 3.2.3. Mortar Fluidity

**Figure 8:**
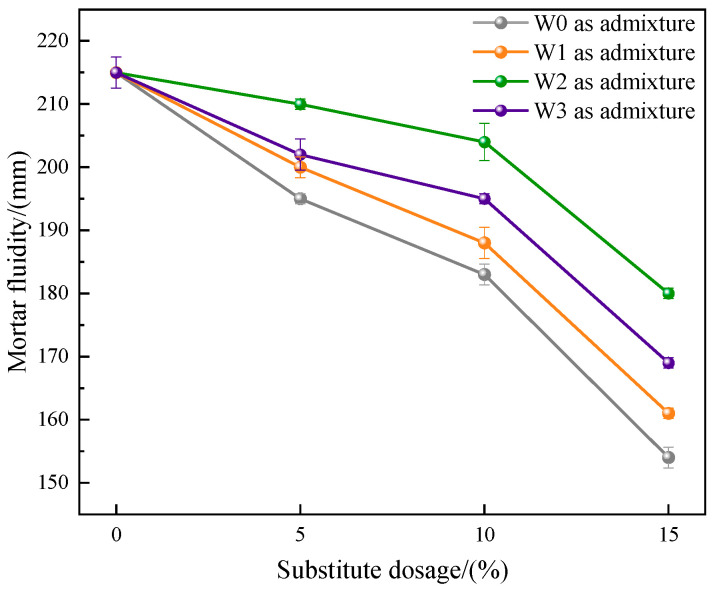
The mortar fluidity of the prepared cement.

The results of mortar fluidity of cement prepared by different admixtures are shown in Figure 8. Among them, the mortar fluidity of the control group was the largest, which was 215 mm. After the incorporation of water-washed incineration fly ash, the mortar fluidity of the cement decreased significantly. With the increase of dosage, the fluidity showed a downward trend, and the minimum fluidity was only 154 mm. This showed that the incorporation of fly ash would cause serious fluidity loss of cement mortar. The reason was that the large specific surface area of fly ash was easy to adsorb water molecules. At the same dosage, compared with the mortar fluidity of cement prepared by the untreated fly ash as admixture, the mortar fluidity of cement prepared by fly ash heat-treated in three atmospheres as admixture increased, and the mortar fluidity of cement prepared by fly ash heat-treated in CO_2_ atmosphere as admixture was the largest. It could be concluded that heat treatment could increase the mortar fluidity, and the improvement effect after heat treatment in CO_2_ atmosphere was more obvious. This was also because the surface morphology of the particles had been more effectively improved after heat treatment of fly ash in CO_2_ atmosphere.

#### 3.2.4. Strength

**Figure 9:**
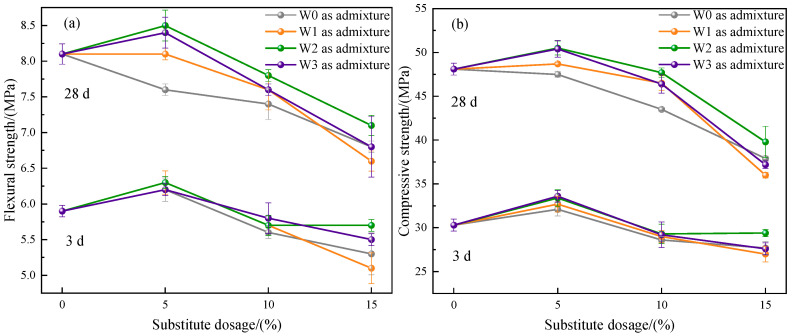
The mortar strength of the prepared cement at different ages. (**a**) Flexural strength; (**b**) Compressive strength.

The results of mortar strength of cement prepared by different admixtures are shown in Figure 9, including flexural strength and compressive strength. For the strength of mortar at 3 d, the flexural and compressive strength of the control group were 5.9 MPa and 30.3 MPa, respectively. After adding water-washed incineration fly ash, when the dosage was 5%, the flexural and compressive strength of the cement were improved, indicating that the addition of a small amount of fly ash had a certain early strength effect on the cement. However, since the activity of fly ash was less than that of cement clinker, when the dosage of fly ash continued to increase, the active substances involved in the hydration reaction were further reduced, resulting in a gradual decrease in the mortar strength of the prepared cement.

For the strength of mortar at 28 d, the flexural and compressive strength of the control group were 8.1 MPa and 48.1 MPa, respectively. When the dosage was 5%, the strength of cement prepared by the untreated fly ash as admixture was lower than that of the control group, while the strengths of cement prepared by fly ash after heat treatment in three atmospheres were higher than that of the control group. At the same dosage, compared with the mortar strength of cement prepared by the untreated fly ash as admixture, the mortar strength of cement was improved by using fly ash as admixture after heat treatment in three atmospheres, and the mortar strength of cement prepared by using fly ash after heat treatment in CO_2_ atmosphere was the largest. This showed that the heat treatment in CO_2_ atmosphere had the best effect of improving the strength of cement mortar prepared by fly ash as admixture. However, when the dosage was 15%, the compressive strength of W1-3, W2-3, and W3-3 was reduced to 36.0 MPa, 39.8 MPa, and 37.2 MPa, respectively. Compared with the control group, it was reduced by 25.16%, 17.26%, and 22.66%, respectively. The reduction was large and could not meet the requirements of P•O42.5 Portland cement strength. Therefore, the dosage of fly ash after heat treatment used as admixture should not exceed 10%.

#### 3.2.5. Hydration Products

**Figure 10:**
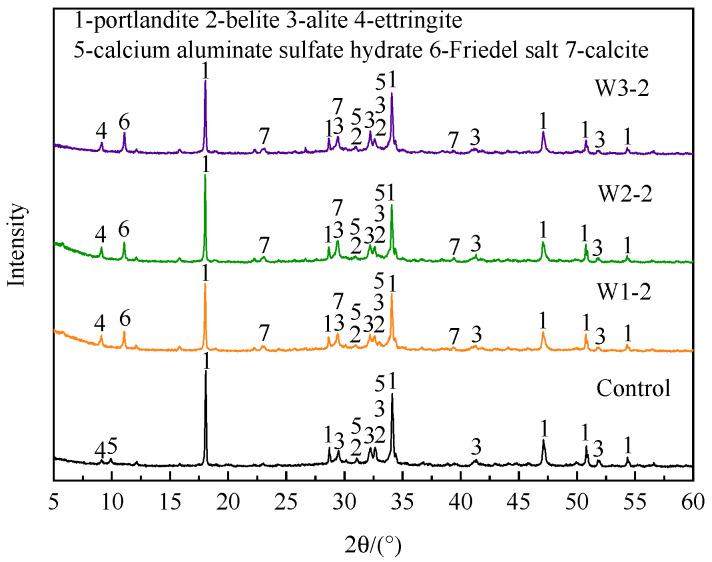
XRD analysis results of 3 d hydration products of the prepared cement.

The XRD analysis results of 3 d hydration products of the control group, W1-2, W2-2, and W3-2 cement, are shown in Figure 10. When the cement hydration age was 3 d, the hydration products of the control group were mainly portlandite (Ca(OH)_2_), ettringite (3CaO•Al_2_O_3_•3CaSO_4_•32H_2_O, AFt), and calcium aluminate sulfate hydrate (3CaO•Al_2_O_3_•CaSO_4_•12H_2_O, AFm), as well as the unhydrated alite (3CaO•SiO_2_, C_3_S) and belite (3CaO•SiO_2_, C_2_S). After adding fly ash, the XRD analysis results of cement hydration products added the diffraction peaks of calcite (CaCO_3_) and Friedel salt (3CaO•Al_2_O_3_•CaCl_2_•10H_2_O) in addition to the above mineral phases. It can be seen in Figure 4 that for calcite, fly ash itself contained calcite. For Friedel salt, it might be due to the reaction of chloride salt in fly ash with cement hydration products to form Friedel salt. Studies have shown that chloride ions interacted with cement hydration products to form Friedel salt [32]. In addition, compared with W1 and W3, there was almost no portlandite in the mineral phase of W2, but the strengths of the diffraction peaks of portlandite in the hydration products of W1-2, W2-2, and W3-2 cement were not much different, indicating that the hydration reaction of W2-2 cement generated more Ca(OH)_2_, and the degree of hydration reaction was higher, resulting in higher mortar strength.

#### 3.2.6. Leaching Characteristics of Heavy Metals in Cements

The mortar specimens of cement prepared by adding 15% fly ash (maximum dosage) were selected. After standard curing to 28 days at a temperature of (20 ± 1) °C and a relative humidity of not less than 90%, the heavy metal leaching concentration of cement mortar specimens was tested by horizontal oscillation method and acetic acid buffer solution method. The results are shown in Table 9 and Table 10. When the dosage of fly ash was the largest, the leaching concentration of heavy metals in the prepared cement mortar specimens was far less than the limit specified in the standard, whether in neutral environment or weak acid environment. It showed that when the dosage of fly ash was 15% or less, the prepared cement did not have the risk of excessive heavy metal leaching. This was because after 28 days of cement hydration, the entire cement body was alkaline, which would promote the precipitation of heavy metals, and the hydrated calcium silicate gel and AFt crystals in the hydration products would also adsorb and encapsulate heavy metals [33], thereby reducing the leaching concentration of heavy metals.

## 4. Conclusions

In this study, the water-washed incineration fly ash was heat-treated in air, CO_2_, and N_2_ atmospheres, and the changes in the physical and chemical properties of the fly ash were systematically studied. Following that, the fly ash heat-treated at the highest carbon fixation temperature (500 °C) was selected as admixture to prepare ordinary Portland cement. The effect of fly ash on cement properties and the possibility of resource utilization of fly ash were studied. The following conclusions were drawn.

1. The mass of fly ash increased after heat treatment in CO_2_ atmosphere. When the temperature was 500 °C, the weight gain reached the maximum, and almost all Ca(OH)_2_ in fly ash was carbonized into CaCO_3_. It could also be observed in SEM that the surface of fly ash formed tightly packed CaCO_3_ crystals. This proved that fly ash had the ability to store CO_2_.
2. The toxic equivalent quantities of dioxins in fly ash after heat treatment (500 °C + 1 h) in air, CO_2_, and N_2_ atmospheres were 17.12 ng TEQ/kg, 0.25 ng TEQ/kg, and 0.14 ng TEQ/kg, respectively, and the degradation rates were 69.95%, 99.56%, and 99.75%, respectively.
3. Compared with the control group without admixture, the water consumption of cement standard consistency increased, and the fluidity of mortar decreased after adding fly ash that was heat-treated in three atmospheres, but the changes of fly ash after heat treatment in CO_2_ atmosphere were the smallest.
4. Compared with the untreated fly ash used as admixture, under the same dosage, when fly ash was used as admixture after heat treatment, the water consumption of cement standard consistency was reduced, the fluidity of mortar was increased, and the strength of 28 d mortar was obviously improved. The changes of fly ash after heat treatment in CO_2_ atmosphere were the most obvious.
5. The fly ash after heat treatment in CO_2_ atmosphere could be used as cement admixture for resource utilization. On the one hand, dioxins were effectively degraded, and the prepared cement had no risk of heavy metal leaching. On the other hand, when the dosage was 10% or less, the performance of the prepared cement met the requirements of P•O42.5 Portland cement.

## Figures and Tables

**Table 1:** Chemical composition of water-washed MSWI FA (wt.%; LOI = loss on ignition).

Chemical Composition	Water-Washed MSWI FA
CaO	48.21
SO_3_	8.54
SiO_2_	6.59
Fe_2_O_3_	2.42
K_2_O	2.03
MgO	2.00
Al_2_O_3_	1.81
Na_2_O	1.49
ZnO	0.61
Others	6.71
LOI (950 °C)	19.59

**Table 2:** Heavy metal contents of water-washed MSWI FA (mg/L).

Heavy Metal	Water-Washed MSWI FA
Zn	3212.19
Pb	312.95
Cd	133.80
Cu	116.07
As	74.16
Cr	72.68
Sb	68.96
Ni	51.46
V	18.49
Co	14.78
Mo	13.85
Tl	4.87
Be	0.54
Hg	0.25

**Table 3:** Chemical composition of Portland cement clinker and natural dihydrate gypsum (wt.%; LOI = loss on ignition).

Chemical Composition	Portland Cement Clinker	Natural Dihydrate Gypsum
CaO	63.60	49.95
SO_3_	0.29	45.58
SiO_2_	23.02	1.56
Al_2_O_3_	5.32	0.68
Fe_2_O_3_	3.46	0.40
K_2_O	1.03	0.07
MgO	1.53	0.35
Others	0.85	1.41
LOI (950 °C)	0.90	ND

Note: “ND” means not detected.

**Table 4:** The formula of the prepared cement (wt.%).

Sequence Number	Water-Washed MSWI FA	Portland Cement Clinker	Natural Dihydrate Gypsum
Control	0	95	5
W0/W1/W2/W3-1	5	90	5
W0/W1/W2/W3-2	10	85	5
W0/W1/W2/W3-3	15	80	5

Note: W0 means the untreated water-washed MSWI FA, W1 means the water-washed MSWI FA after heat treatment at 500 °C in air, W2 means the water-washed MSWI FA after heat treatment at 500 °C in CO_2_, and W3 means the water-washed MSWI FA after heat treatment at 500 °C in N_2_. W0-1, W0-2, and W0-3, respectively, mean dosage of the untreated water-washed MSWI FA at 5%, 10%, and 15%. Other analogies.

**Table 5:** Key parameters of particle size distribution.

Category	D10 (μm)	D50 (μm)	D90 (μm)	Dispersion
W1	1.66	17.78	112.81	6.25
W2	1.35	30.00	144.10	4.76
W3	1.48	22.21	138.74	6.18

**Table 6:** Heavy metal leaching concentration of fly ash before and after heat treatment in different atmospheres (mg/L), using the horizontal oscillation method.

Heavy Metal	W0	W1	W2	W3	Landfill Limit
Cr	0.2728	0.0763	0.0824	0.1255	4.5
Ni	0.0186	0.0102	0.0053	0.0108	0.5
Cu	0.0249	0.0581	0.0297	0.0345	40
Zn	0.2049	0.1434	0.1562	0.1862	100
Cd	0.0007	0.0002	0.0002	0.0004	0.15
Pb	0.0476	0.0866	0.0321	0.0847	0.25

**Table 7:** Heavy metal leaching concentration of fly ash before and after heat treatment in different atmospheres (mg/L), using the acetic acid buffer solution method.

Heavy Metal	W0	W1	W2	W3	Landfill Limit
Cr	0.4786	0.2877	0.3190	0.1310	4.5
Ni	0.0761	0.0444	0.0671	0.0410	0.5
Cu	0.1672	0.3160	0.2609	0.2754	40
Zn	11.3309	7.0022	6.0715	7.9853	100
Cd	0.0850	0.0469	0.0327	0.0565	0.15
Pb	0.3187	0.2879	0.1298	0.2658	0.25

**Table 8:** Toxic equivalent quantities of fly ash before and after heat treatment in different atmospheres (pg/kg).

Isomer	TEF	W0		W1		W2		W3	
		C	TEQ	C	TEQ	C	TEQ	C	TEQ
2,3,7,8-T_4_CDD	1	650	650	440	440	100	100	32	32
1,2,3,7,8-P_5_CDD	0.5	420	210	270	135	124	62	96	48
1,2,3,4,7,8-H_6_CDD	0.1	11,000	1100	4550	455	30	3	25	2.50
1,2,3,6,7,8-H_6_CDD	0.1	18,000	1800	8350	835	23	2.30	20	2
1,2,3,7,8,9-H_6_CDD	0.1	12,000	1200	6380	638	21	2.10	20	2
1,2,3,4,6,7,8-H_7_CDD	0.01	180,000	1800	66,700	667	60	0.60	41	0.41
O_8_CDD	0.001	470,000	470	135,000	135	4300	4.30	180	0.18
2,3,7,8-T_4_CDF	0.1	28,000	2800	8000	800	120	12	100	10
1,2,3,7,8-P_5_CDF	0.05	48,000	2400	8700	435	66	3.30	42	2.10
2,3,4,7,8-P_5_CDF	0.5	56,000	28,000	15,844	7922	70	35	36	18
1,2,3,4,7,8-H_6_CDF	0.1	38,000	3800	9400	940	55	5.50	42	4.20
1,2,3,6,7,8-H_6_CDF	0.1	49,000	4900	11,240	1124	60	6	42	4.20
1,2,3,7,8,9-H_6_CDF	0.1	13,000	1300	2670	267	80	8	65	6.50
2,3,4,6,7,8-H_6_CDF	0.1	49,000	4900	18,770	1877	55	5.50	48	4.80
1,2,3,4,6,7,8-H_7_CDF	0.01	140,000	1400	37,500	375	39	0.39	640	6.40
1,2,3,4,7,8,9-H_7_CDF	0.01	17,000	170	4500	45	60	0.60	24	0.24
O_8_CDF	0.001	73,000	73	30,000	30	550	0.55	340	0.34
TEQ	-	-	56,973	-	17,120	-	251.14	-	143.87

Note: TEQ means toxic equivalent quantity, TEF means toxic equivalent factor, “C” means concentration, “-” means that the parameter is not obtained.

**Table 9:** Heavy metal leaching concentration of the prepared cement (mg/L) using the horizontal oscillation method.

Heavy Metal	W0-3	W1-3	W2-3	W3-3	Landfill Limit
Cr	0.0663	0.0255	0.0038	ND	4.5
Ni	0.0015	0.0045	0.0035	0.0042	0.5
Cu	ND	ND	ND	ND	40
Zn	0.0065	ND	0.0111	0.0031	100
Cd	0.0001	0.0001	ND	ND	0.15
Pb	0.0014	0.0018	0.0010	0.0026	0.25

Note: “ND” means not detected.

**Table 10:** Heavy metal leaching concentration of the prepared cement (mg/L) using the acetic acid buffer solution method.

Heavy Metal	W0-3	W1-3	W2-3	W3-3	Landfill Limit
Cr	0.0705	0.0449	0.0602	0.0450	4.5
Ni	0.0192	0.0134	0.0314	0.0143	0.5
Cu	0.0691	0.0592	0.0697	0.0787	40
Zn	0.7283	0.7137	0.5463	0.5767	100
Cd	0.0121	0.0199	0.0136	0.0149	0.15
Pb	0.0548	0.0808	0.0540	0.0450	0.25

## Data Availability

Not applicable.
